# Exposure of zebra mussels to radial extracorporeal shock waves: implications for treatment of fracture nonunions

**DOI:** 10.1186/s13018-021-02852-1

**Published:** 2021-12-04

**Authors:** Wenkai Wu, Nicola Maffulli, John P. Furia, Lukas Meindlhumer, Katharina Sternecker, Stefan Milz, Christoph Schmitz

**Affiliations:** 1 Extracorporeal Shock Wave Research Unit, Chair of Neuroanatomy, Institute of Anatomy, Faculty of Medicine, LMU Munich, 80336 Munich, Germany; 2 Department of Musculoskeletal Disorders, Faculty of Medicine and Surgery, University of Salerno, Salerno, Italy; 3 Guy Hilton Research Centre, School of Pharmacy and Bioengineering, Keele University, Stoke-on-Trent, Staffordshire ST4 7QB England, UK; 4 Centre for Sports and Exercise Medicine, Barts and The London School of Medicine and Dentistry, Mile End Hospital, London, E1 4DG England, UK; 5 SUN Orthopedics of Evangelical Community Hospital, 210 JPM Rd, Lewisburg, PA 17837 USA

**Keywords:** rESWT, Biomineralization, Mussel shell, Calcein green, Fracture non-union

## Abstract

**Background:**

Radial extracorporeal shock wave therapy (rESWT) is an attractive, non-invasive therapy option to manage fracture nonunions of superficial bones, with a reported success rate of approximately 75%. Using zebra mussels (*Dreissena polymorpha*), we recently demonstrated that induction of biomineralization after exposure to focused extracorporeal shock waves (fESWs) is not restricted to the region of direct energy transfer into calcified tissue. This study tested the hypothesis that radial extracorporeal shock waves (rESWs) also induce biomineralization in regions not directly exposed to the shock wave energy in zebra mussels.

**Methods:**

Zebra mussels were exposed on the left valve to 1000 rESWs at different air pressure (between 0 and 4 bar), followed by incubation in calcein solution for 24 h. Biomineralization was evaluated by investigating the fluorescence signal intensity found on sections of the left and right valves prepared two weeks after exposure.

**Results:**

General linear model analysis demonstrated statistically significant (*p* < 0.05) effects of the applied shock wave energy as well as of the side (left/exposed vs. right/unexposed) and the investigated region of the valve (at the position of exposure vs. positions at a distance to the exposure) on the mean fluorescence signal intensity values, as well as statistically significant combined energy × region and energy × side × region effects. The highest mean fluorescence signal intensity value was found next to the umbo, i.e., not at the position of direct exposure to rESWs.

**Conclusions:**

As in the application of fESWs, induction of biomineralization by exposure to rESWs may not be restricted to the region of direct energy transfer into calcified tissue. Furthermore, the results of this study may contribute to better understand why the application of higher energy flux densities beyond a certain threshold does not necessarily lead to higher success rates when treating fracture nonunions with extracorporeal shock wave therapy.

## Background

Extracorporeal shock wave therapy (ESWT) has become an attractive, non-invasive option for the management of fracture nonunions [1–3]. Current treatment protocols recommend exact application of focused extracorporeal shock waves (fESWs) at the fracture line with the highest possible energy flux density (EFD) [4, 5]. This requires high effort and large, stationary and expensive focused ESWT (fESWT) devices. On the other hand, recent reports described successful treatment of fracture nonunions of superficial bones using radial ESWT (rESWT) [3, 6], in line with what was obtained in animal models [7, 8], with a reported success rate of approximately 75% [3, 6].

Both fESWs and radial extracorporeal shock waves (rESWs) are single acoustic impulses which have an initial high positive peak pressure between 10 and more than 100 megapascals that is reached in less than one microsecond (µs) [9, 10]. The positive pressure is followed by a low tensile amplitude of a few microseconds duration that can generate cavitation [11]. The life cycle of single fESWs or rESWs is approximately 10–20 μs [9–11]. Given these characteristics, fESWs and rESWs fundamentally differ from therapeutic ultrasound. Furthermore, fESWs differ from rESWs in terms of how the shock waves are produced, with regard to the penetration depth of the shock waves into tissue, and in terms of their physical characteristics [9, 11, 12].

Prior studies [3, 6] indicated that rESWT could become a highly attractive alternative to fESWT in the management of fracture nonunions of superficial bone (including the tibia, fibula, bones of the hand and foot, clavicle, etc.). rESWT as opposed to fESWT might be advantageous, as the former is less expensive and does not require exact application at the fracture line (and, thus, not exact positioning using, e.g., an image intensifier). Furthermore, treatment with rESWs is usually less painful than treatment with fESWs and does not require local anesthesia or sedation [12]. Further, rESWT devices are more widely used than fESWT devices, and there is no scientific evidence in favor of either rESWT or fESWT in terms of treatment outcome when treating tendon and other pathologies of the musculoskeletal system [12].

Using zebra mussels (*Dreissena polymorpha*) as a model for studying biomineralization [13], we recently demonstrated that induction of biomineralization after exposure to fESWs is not restricted to the region of direct energy transfer into calcified tissue [10] (detection of newly calcified tissue was performed by exposing the mussels to fluorescent markers that were incorporated into the shell during biomineralization). It is currently unknown whether this is also true for rESWs.

Accordingly, this study aimed to test the following hypotheses: (1) as fESWs, rESWs also induce biomineralization in zebra mussels; and (2) there is a direct dose-dependent effect in the formation of newly calcified tissue after exposure of zebra mussels to rESWs (i.e., "the more the better").

## Methods

### Animals

The data presented in this paper were produced in two experiments performed in 2018 (*n* = 60 mussels) and 2019 (*n* = 30 mussels). Zebra mussels *(Dreissena polymorpha*) were collected by hand from the rivers Götzinger Ache (Bavaria, Germany) in March 2018 and Schinderbach (Bavaria, Germany) in July 2019. The mussels were fed ad libitum with shellfish diet in 2018 and with *Chlorella vulgaris* (SAG Number 211-19; Algae collection of the University of Goettingen, Goettingen, Germany) in 2019 before and during the experiments. The mussel size was measured before sacrificing according to [14] (mean length 23 ± 2.2 mm (mean ± standard deviation); width, 12 ± 1.5 mm; height 11 ± 1.2 mm).

All experiments were performed according to German animal protection regulations which do not require registration or approval of experiments using zebra mussels.

### Exposure of mussels to radial extracorporeal shock waves

**Fig. 1:**
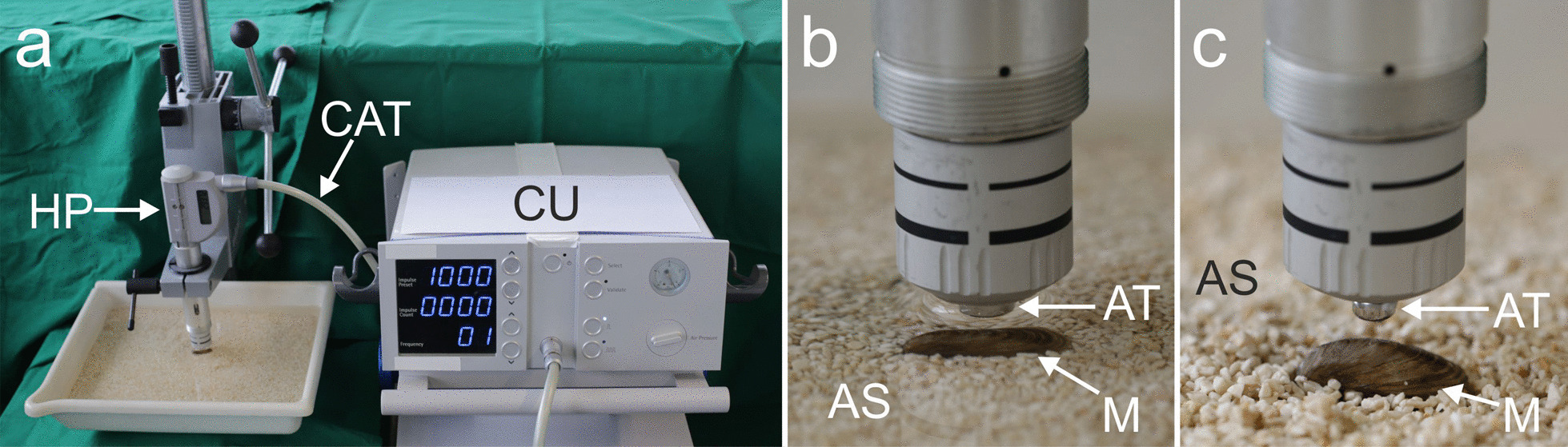
Exposure of Zebra mussels to radial extracorporeal shock waves. **a** overview; **b** close-up view of the mussel and the metal applicator of the handpiece of the radial extracorporeal shock wave therapy (rESWT) device under water; **c** close-up view as in **b** but without water, showing the distance between the mussel and the applicator of the handpiece of the rESWT device. Abbreviations: CU, control unit; CAT, compressed-air tube; HP, handpiece; AT, applicator tip; M, mussel; AS, aquarium sand

**Fig. 2:**
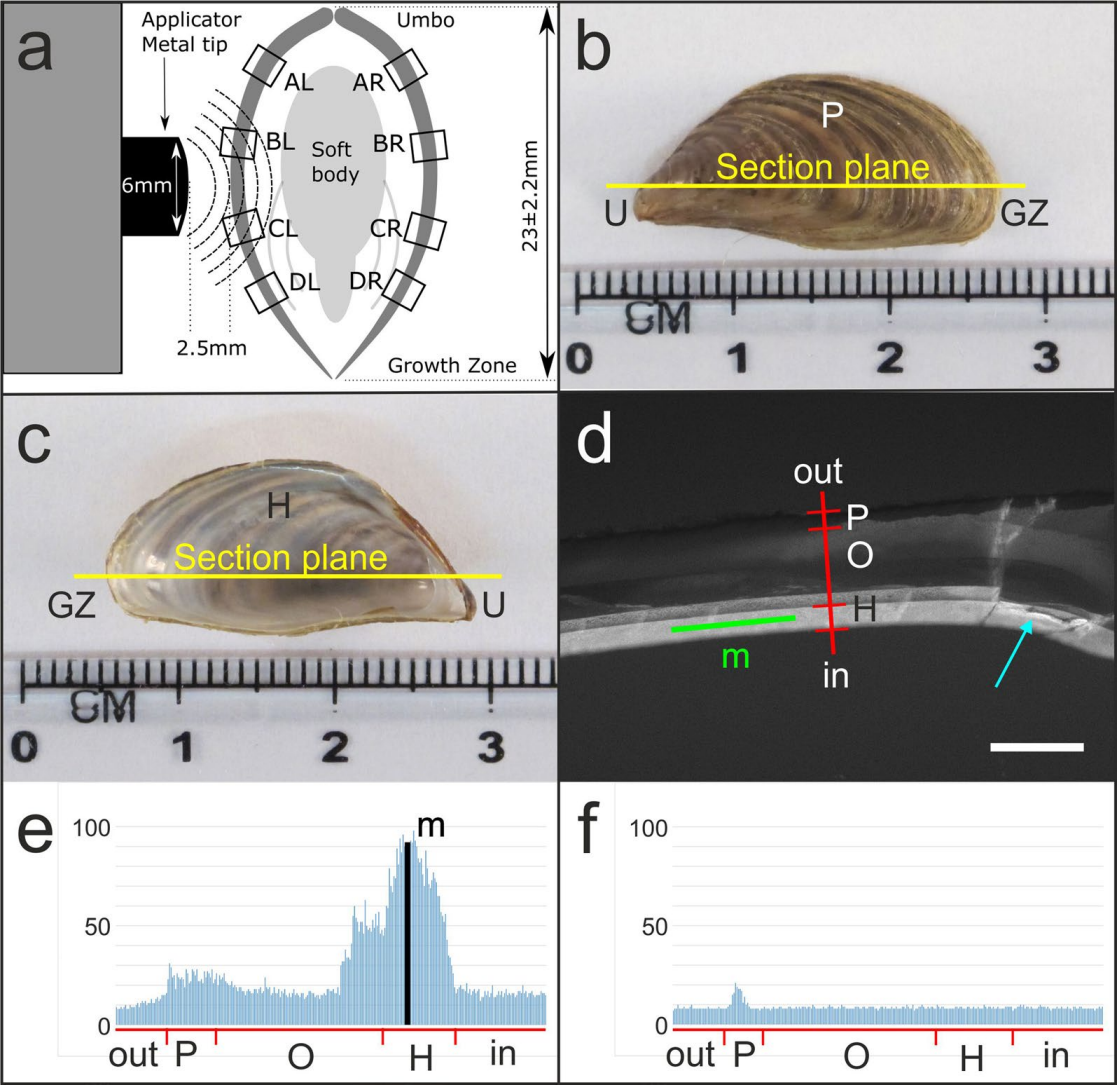
Principle of exposing zebra mussels to radial extracorporeal shock waves (rESWs) and analyzing the effects on biomineralization. **a** Schematic of a cross section through a zebra mussel, indication of Regions A–D on the left (AL-DL) and the right (AR-DR) valve, and sketch of the metal applicator of the handpiece of the radial extracorporeal shock wave therapy device true to scale. **b**, **c** Side view on the valve of a zebra mussel from outside (**b**) and inside (**c**). The section plane is indicated. **d** Principle of investigating the formation of new mineralized tissue after exposure of zebra mussels to rESWs using fluorescence microscopy by determining the fluorescence signal intensity (Calcein fluorescence imaging) along the indicated green line over the hypostracum. The blue arrow indicates an artifact that was caused by the methodology used for generating the sections, resulting in irregular fluorescence signal intensity. **e**, **f** Representative linear pixel plots of the fluorescence signal intensity (in arbitrary units) along the red line shown in **d**, demonstrating high fluorescence signal intensity values specifically over the hypostracum after exposure to rESWs produced at 3.5 bar (**e**) but not after sham exposure (**f**). Abbreviations: U, umbo; P, periostracum; H, hypostracum; GZ, growth zone; out, outside surface of the muscle valve; in, inside surface of the muscle valve; m, maximum fluorescence signal intensity found over the hypostracum. The scale bar in **d** represents 300 µm

The mussels were exposed to rESWs produced with a Swiss DolorClast device (Electro Medical Systems, Nyon, Switzerland), using the radial handpiece and 6-mm applicator (Figs. 1, 2a). During the first/second experiment performed in 2018/2019, *n* = 10/*n* = 5 mussels each were randomly selected and exposed to 1000 rESWs each produced using an air pressure of the rESWT device of, respectively, 0 bar (sham exposure), 2.0, 2.5, 3.0, 3.5 or 4.0 bar.

For exposure to rESWs, the mussels were fixed under water in aquarium sand (diameter 2–3 mm; Dupla Marin Reef Ground; Dohse Aquaristik, Grafschaft-Gelsdorf, Germany) to disperse and, thus, minimize the reflection of rESWs (Fig. 1). Using a drill stand, the distance between the applicator tip and the mussels was set at 2.5 mm to prevent any mechanical destruction of the mussel valve through direct contact with the applicator tip. Accordingly, the energy flux density (EFD) at 3.0 and 4.0 bar air pressure that hit the mussels was approximately 0.08 mJ/mm^2^ and 0.11 mJ/mm^2^ (the EFD generated using the 6 mm applicator of the handpiece of the rESWT device shown in Fig. 1 is similar to the EFD generated using the 15 mm applicator of this device [15]; the decrease in the EFD is almost linear between a distance of 1 mm and 5 mm to the applicator [15]. At a distance of 1 mm and 5 mm to the applicator, the following EFDs were measured using the 15-mm applicator [11]: 0.1 mJ/mm^2^ and 0.04 mJ/mm^2^ when operated at 3.0 bar air pressure, and 0.14 mJ/mm^2^ and 0.06 mJ/mm^2^ when operated at 4.0 bar air pressure). The rESWs were applied at a frequency of 8 Hz.

Immediately after exposure to rESWs or sham exposure, the mussels were incubated in calcein solution (10 mg/l; Product Number: C0875-5G; Sigma-Aldrich, St. Louis, MO, USA) for 24 h. To this end, all mussels were placed in the same aquarium which contained six liters of calcein solution, with each group of mussels in a separate glass chamber (10 × 15 × 15 cm). The position of each glass chamber within the aquarium was selected randomly. Afterwards, the mussels were housed (using the same glass chambers and aquarium) in ventilated tap water for two weeks. Then, the mussels were euthanized in 70% ethanol, and the dissected valves were dehydrated in increasing concentrations of ethanol (70%, 80% and 90% for six days each, followed by 100% for 12 days).

After fixation, both valves of each mussel were degreased in xylene for six days, followed by incubation in methanol for six days. Then, the mussel valves were embedded in methyl methacrylate (Product Number: 800590; Sigma-Aldrich) according to [16]. Polymerization took 14 days. Afterward, the polymerized methyl methacrylate blocks containing the valves (one valve per block) were cut into 400-µm-thick sections along the longest axis of the embedded valve using a ring saw microtome (SP 1600; Leica, Wetzlar, Germany) (Fig. 2b, c). The sections were ground and polished using a 400 CS micro-grinder (EXAKT Advanced Technologies, Norderstedt, Germany). The final section thickness was approximately 200 µm, measured in the middle of each section using a digimatic micrometer (Mitutoyo, Kawasaki, Japan).

### Measurements of fluorescence signal intensity

Images were taken using a fluorescence microscope (Olympus BX51WI; Olympus, Tokyo, Japan) using a 4 × UPlanSApo objective (numerical aperture = 0.16) (Olympus), Alexa Fluor 488 filter (49011; Chroma, Bellows Falls, VT, USA), grayscale EM CCD camera (Model C9100-02, 1000 × 1000 pixels; Hamamatsu Photonics, Hamamatsu City, Japan) and SOLA LED lamp (Lumencor, Beaverton, OR, USA). All images were taken with the Stereo Investigator software (64 bit, Version 11.07; MBF Bioscience, Williston, VT, USA) and saved as 8 bit TIF files (i.e., with gray values ranging from 0 to 255). Using pilot measurements, the camera was adjusted so that no image was overexposed (i.e., all gray values were smaller than 255). This resulted in the following camera settings: exposure time, 24 ms; sensitivity, 80; gamma, 1.0.

In line with our previous study [10], the strongest fluorescence signal was found over the hypostracum (Fig. 2d). Analysis of mussels after sham exposure indicated that the signal over the hypostracum was indeed caused by exposure to rESWs (Fig. 2e, f). Accordingly, measurements of fluorescence signal intensity were performed over the hypostracum, using the linear pixel plot function of the Stereo Investigator software (MBF Bioscience). Four measurement lines each (spanning 243 ± 79 µm representing 135 ± 44 pixels, depending on the curvature of the valve) were positioned over the hypostracum as shown in Fig. 2d, representing Regions A-D indicated in Fig. 2a. Region A was next to the umbo, Region D was next to the shell growth zone, and Regions B and C were in between. As in our previous study [10], the umbo itself was excluded from the analysis because of strong autofluorescence of the ligament.

### Statistical analysis

For each group of mussels (i.e., each intensity of the rESWs) mean and standard deviation of side- and region-specific fluorescence signal intensities were calculated. Outliers were identified using the Tukey's fences method [17] (with *k* > 1.5 indicating an outlier) and removed (outlier values were most probably caused by the methodology used for generating the sections, in particular by grinding and polishing). The corresponding calculations were performed using GraphPad Prism (Version 9.2.0 for Windows; GraphPad Software, San Diego, CA, USA). Fifty-seven out of the 720 individual data (six groups of mussels × 15 mussels per group × two valves per muscle × four regions per valve) (7.9%) were identified as outliers. The absolute and relative numbers of valves with 0/1/2/3/4 outlier values in their respective group were 145/21/9/2/3 and 80.6%/11.7%/5.0%/1.1%/1.7%, respectively. After removal of outliers, there were at least 12 (out of 15 maximally possible) values available for each combination of energy, side and region.

Then, differences in mean fluorescence signal intensities were investigated using general linear model analysis, with energy (i.e., the intensity of the rESWs), side (left/exposed vs. right/unexposed) and region (regions A–D shown in Fig. 2a) as fixed factors and the averaged fluorescence signal intensities (one value each per mussel, side and region) as depending factor. Post hoc analyses (energy, region) were performed using Bonferroni's multiple comparison test. Calculations were performed using SPSS (Version 26.0.0.0; IBM, Armonk, NY, USA). *P* values smaller than 0.05 were considered statistically significant.

## Results

**Fig. 3:**
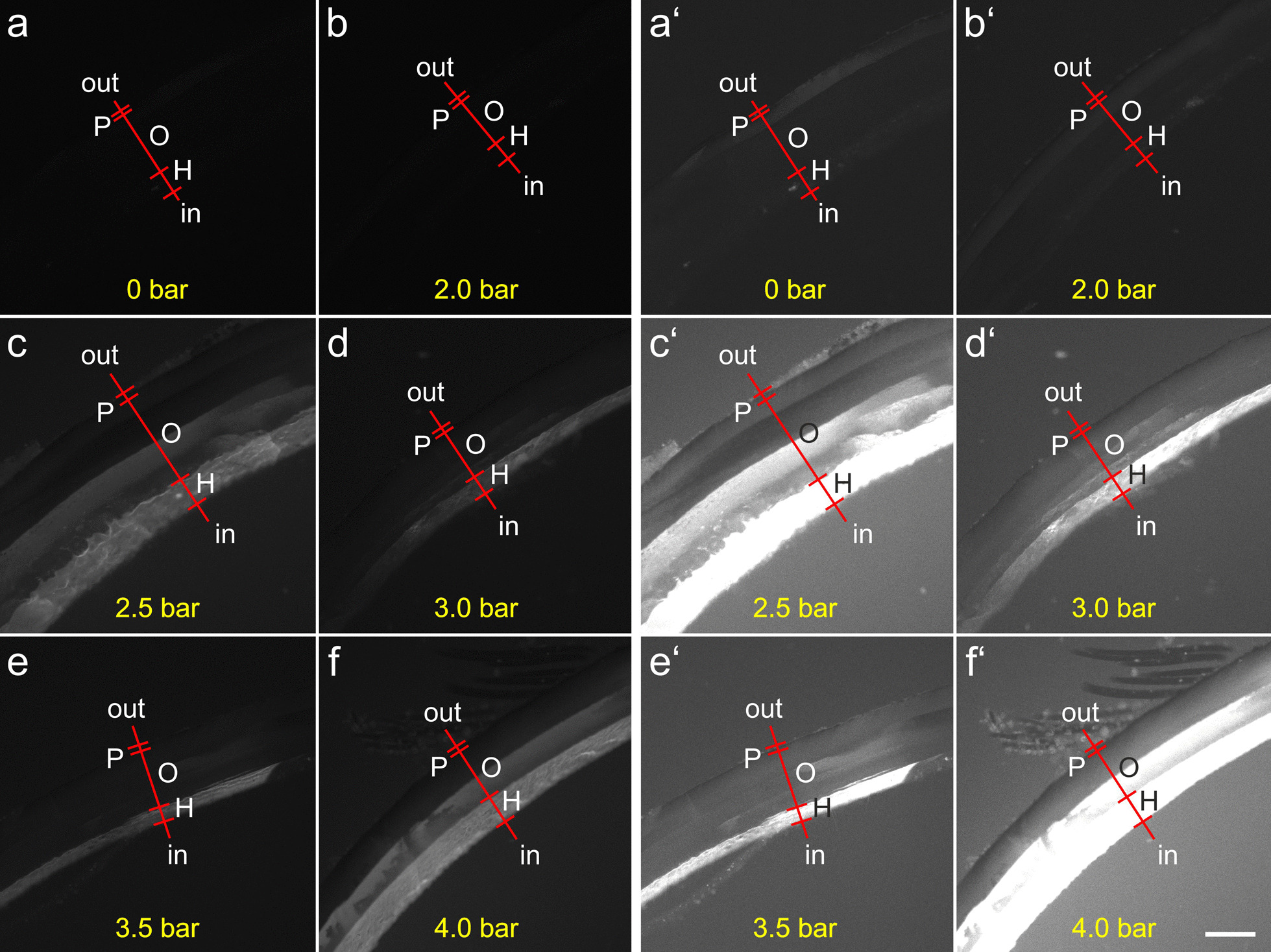
Representative photomicrographs of sections of the left valve of zebra mussels exposed to radial extracorporeal shock waves as shown in Fig. 2a produced with 0 (**a**), 2.0 (**b**), 2.5 (**c**), 3.0 (**d**), 3.5 (**e**) and 4.0 (**f**) bar. In order to correctly identify the individual layers of the valves, the brightness of the images was greatly increased as shown in Panels a' to f'. Abbreviations: out, outside surface of the mussel shell; P, periostracum; O, ostracum; H, hypostracum; in, inside surface of the mussel shell. The scale bar in **f'** represents 300 µm in **a**–**f**

Qualitative analysis of the left (exposed) valves indicated a dose-dependent increase in the fluorescence signal intensity particularly over the hypostracum (Fig. 3).

**Fig. 4:**
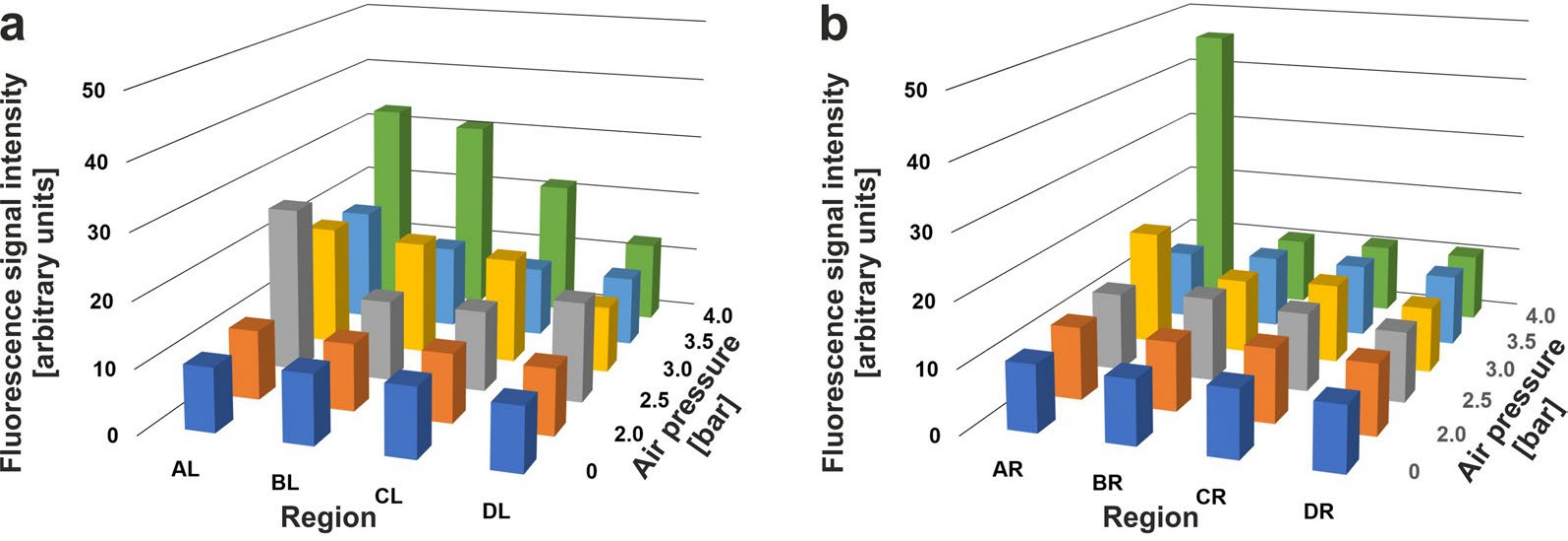
3D histograms of the mean fluorescence signal intensity values found over Regions AL-DL (**a**) and AR-DR (**b**) shown in Fig. 2a as a function of the air pressure (0, 2.0, 2.5, 3.0., 3.5 and 4.0 bar) used to produce the radial extracorporeal shock waves

Table 1 summarizes mean and standard deviation of energy-, side- and region-specific fluorescence signal intensity values; Fig. 4 provides a three-dimensional (3D) graphical representation of the mean values. The results of the statistical analysis are listed in Table 2.

**Table 1:** Mean and standard deviation of energy-, side (left/right)- and region (regions A–D as shown in Fig. 2a)-specific fluorescence signal intensity values (arbitrary units)

Bar	AL	AR	BL	BR	CL	CR	DL	DR
0	9.9 ± 0.8 (14)	10.4 ± 1.3 (15)	10.8 ± 2.1 (15)	10.1 ± 0.8 (14)	10.7 ± 1.0 (15)	10.5 ± 0.9 (14)	9.9 ± 0.6 (15)	10.0 ± 0.7 (15)
2.0	10.8 ± 0.9 (15)	11.3 ± 2.1 (13)	10.3 ± 1.1 (15)	10.7 ± 0.9 (13)	10.6 ± 0.9 (14)	11.5 ± 1.2 (14)	10.1 ± 1.0 (14)	10.9 ± 1.3 (14)
2.5	25.7 ± 22.6 (14)	12.2 ± 3.2 (14)	12.6 ± 2.2 (12)	13.0 ± 4.3 (14)	12.4 ± 2.4 (12)	12.2 ± 2.7 (14)	15.5 ± 7.3 (15)	11.0 ± 1.4 (15)
3.0	18.9 ± 12.8 (14)	18.3 ± 12.0 (15)	17.7 ± 10.0 (14)	11.7 ± 1.7 (13)	16.7 ± 8.0 (15)	12.4 ± 2.8 (13)	10.4 ± 1.2 (13)	10.3 ± 0.7 (12)
3.5	18.3 ± 15.7 (14)	10.9 ± 3.6 (12)	13.2 ± 6.7 (13)	11.5 ± 3.3 (12)	11.1 ± 2.2 (12)	11.5 ± 3.1 (13)	10.8 ± 1.9 (13)	11.3 ± 2.9 (14)
4.0	32.8 ± 32.6 (15)	45.5 ± 51.3 (15)	30.9 ± 30.7 (14)	10.7 ± 1.3 (12)	22.4 ± 19.1 (13)	10.9 ± 0.9 (14)	12.7 ± 3.5 (14)	10.7 ± 0.9 (15)

The numbers in parentheses indicate the number of values per group after removal of outliers.

L, left; R, right

**Table 2:** Outcome (*P* values) of the statistical analysis of the data shown in Table 1

Results of general linear model analysis	*P*
Energy	** < 0.001**
Side	**0.018**
Region	** < 0.001**
Energy × side	0.462
Energy × region	** < 0.001**
Side × region	0.509
Energy × side × region	**0.005**

Region A		Region B		Region C	
Comparison with	*P*	Comparison with	*P*	Comparison with	*P*
*Results of post hoc Bonferroni tests comparing different regions (A–D, shown in *Fig. 2a) *with each other*					
Region B	** < 0.001**				
Region C	** < 0.001**	Region C	1.000		
Region D	** < 0.001**	Region D	0.399	Region D	1.000

4 bar		3.5 bar		3.0 bar		2.5 bar		2.0 bar	
Comparison with	*P*	Comparison with	*P*	Comparison with	*P*	Comparison with	*P*	Comparison with	*P*
*Results of post hoc Bonferroni test comparing different energy settings with each other*									
3,5 bar	** < 0.001**								
3 bar	** < 0.001**	3 bar	1.000						
2,5 bar	** < 0.001**	2.5 bar	1.000	2.5 bar	1.000				
2 bar	** < 0.001**	2 bar	1.000	2 bar	0.278	2 bar	0.478		
0 bar	** < 0.001**	0 bar	1.000	0 bar	0.100	0 bar	0.185	0 bar	1.000

*P* < 0.05 are given boldface

Figure 4 indicates energy-, side- and region-specific differences in mean fluorescence signal intensity values. In line with this, general linear model analysis demonstrated statistically significant effects of the applied shock wave energy (*p* < 0.001) as well as of the side (*p* = 0.018) and the investigated region (*p* < 0.001) on the fluorescence signal intensity values, as well as statistically significant combined energy × region (*p* < 0.001) and energy × side × region (*p* = 0.005) effects (Table 2). The highest mean fluorescence signal intensity values were found in Region A, i.e., next to the umbo (Table 1). Post hoc Bonferroni tests demonstrated statistically significant differences between the mean fluorescence signal intensity values measured in Region A compared to the mean fluorescence signal intensity values measured in all other regions, but no statistically significant differences between the mean fluorescence signal intensity values measured in Regions B, C and D (Table 2). Furthermore, post hoc Bonferroni tests demonstrated statistically significant differences between the mean fluorescence signal intensity values obtained after exposure of mussels to rESWs produced at 4.0 bar and the mean fluorescence signal intensity values obtained after exposure to rESWs produced at, respectively, 0, 2.0, 2.5, 3.0 and 3.5 bar, but no statistically significant differences between mean fluorescence signal intensity values obtained after exposure to rESWs produced at respectively 0, 2.0, 2.5, 3.0 or 3.5 bar (Table 2).

## Discussion

This study demonstrated that exposure of zebra mussels to rESWs had an effect on the biomineralization of the mussel valve, in a complex, dose- and region-specific manner.

The decrease in the mean fluorescence signal intensity values from the umbo (Region A) to the growth zone (Region D) found in this study was in line with earlier results obtained after exposure of zebra mussels to fESWs [10], representing the physiological mineralization process of mussel shells [18, 19]. The increased fluorescence signal intensity after exposure to rESWs was detected over the hypostracum, i.e., the shell layer which reacts with increased biomineralization after shell injuries [20, 21].

On the other hand, there was a substantial difference between the results of this study (exposure to rESWs) and our earlier study (exposure to fESWs). Specifically, after exposure of zebra mussels to fESWs, no statistically significant difference was found in the mean fluorescence signal intensity values between the exposed (left) and unexposed (right) valves [10], which was different in this study (Fig. 4; Table 2). This was most probably caused by differences in the applied shock wave energy: exposure to fESWs was performed with EFD = 0.4 mJ/mm^2^ in [10], whereas, in the present study, the highest EFD was approximately 0.11 mJ/mm^2^. Thus, the lower EFD of the rESWs applied in this study was likely too low to result in a similar biological reaction (i.e., induction of biomineralization in both the exposed and unexposed valves) than the much higher EFD of the fESWs applied in our previous investigation [10]. In that study, even though the fESW energy could not reach the unexposed mussel valve, a biological reaction on both sides was triggered, probably caused by the high EFD of the fESWs applied [10]. In the present study, the energy of the rESWs was apparently high enough to activate cells of the shell epithelium to induce biomineralization in the exposed valve. However, the energy was probably too low to activate cells inside the soft body, e.g., the hemocytes carrying crystals or the crystal formation related cells [21–23]. This will be addressed in detail in future studies.

The following, unexpected results of this study could not be explained. First, the highest mean fluorescence signal intensity values were found in Region AR (i.e., on the unexposed valve) after exposure of the mussels to rESWs produced at 4.0 bar air pressure. One possible explanation was the proximity of this region to the umbo. (Note that on the exposed valve the highest mean fluorescence signal intensity values were also found in Region A.)

**Fig. 5:**
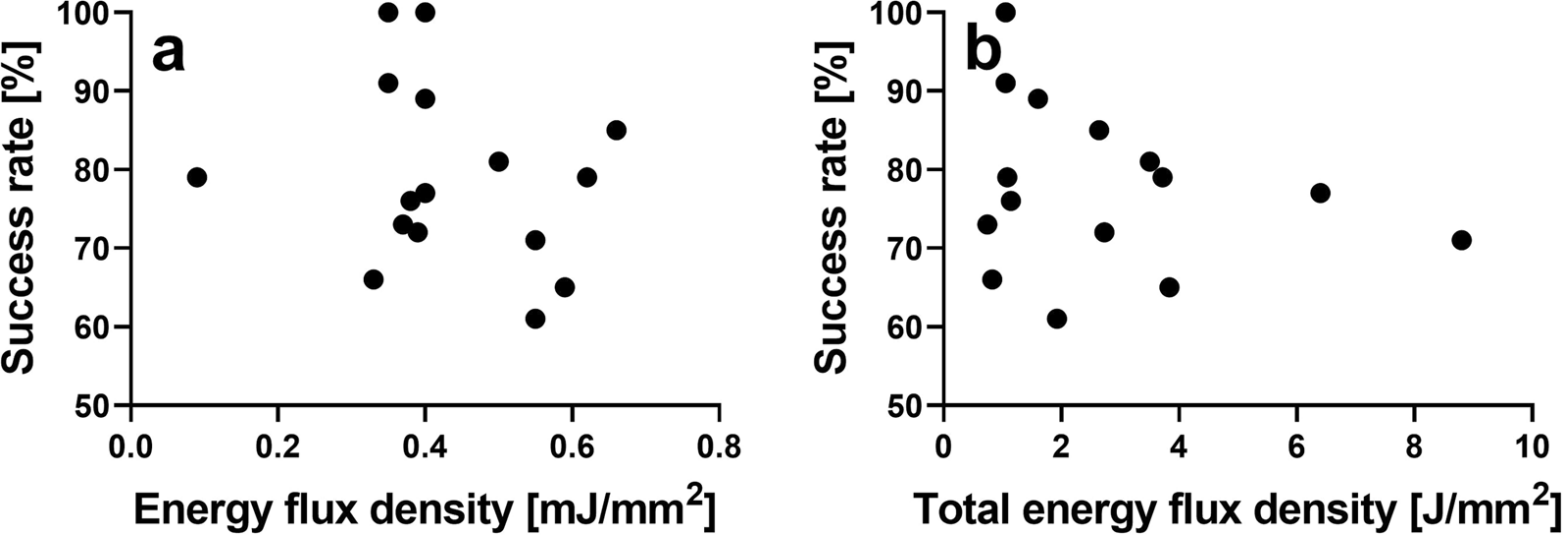
Success rate (defined as the relative number of patients with radiographic union confirmed six months post focused extracorporeal shock wave therapy (fESWT)) as a function of the energy flux density (EFD) of individual focused extracorporeal shock waves (fESWs) (**a**) as well as the total EFD (individual EFD multiplied with the number of applied fESWs per treatment session and the number of treatment sessions) (**b**) in those 16 clinical studies on fESWT for treating fracture nonunions listed in Table 1 in [3] for which both the EFD of the applied fESWs and the success rate were reported

Second, almost no difference in the mean fluorescence signal intensity values was observed at Region AL between mussels exposed to rESWs produced at respectively 0 bar (sham exposure) or 2.0 bar air pressure, whereas the valves of mussels exposed to rESWs produced at 2.5 bar air pressure showed a much higher mean fluorescence signal intensity value at Region AL. The latter even exceeded the mean fluorescence signal intensity values at Region AL of those mussels which were exposed to rESWs produced at, respectively, 3.0 bar and 3.5 bar. The reason for this phenomenon, which occurred independently in both experiments performed in 2018 and 2019 (details not shown), is unknown. In any case, this phenomenon could indicate, for the first time, that there is no direct relationship between the applied EFD of extracorporeal shock waves (ESWs) and the extent of biomineralization in the target tissue. In this regard, it is of note that there was no direct relationship between the EFD of the applied fESWs and the success rate (defined as the relative number of patients with radiographic union confirmed six months post fESWT) in those 16 clinical studies on fESWT for treating fracture nonunions listed in Table 1 in [3] for which both the EFD of the applied fESWs and the success rate were reported (Fig. 5). Taken together, the results of this study may provide a reason for the phenomenon shown in Fig. 5, combined with the insight that higher EFDs beyond a certain threshold do not necessarily lead to higher success rates in treatments of fracture nonunions using ESWT. Further investigation of this phenomenon may be difficult using vertebrate animal models, considering the high number of animals which would be required. As such, exposure of zebra mussels to rESWs (as well as to fESWs) may become an attractive animal model in future research into the molecular and cellular mechanisms of ESWs in the management of fracture nonunions under consideration of the principles of the 3Rs (Replacement, Reduction, and Refinement) in research involving animal models.

### Limitations

This study has several limitations. One limitation is the use of a non-vertebrate animal model in research focusing on treatments of bone injuries. However, the principles of biocalcification in invertebrates with calcified tissues, particularly mussels, show, despite their different mineral types, many similarities to those observed in vertebrate bone (details are provided in [10]). Another limitation is that this study did not contribute to better understand the molecular and cellular mechanisms of ESWs in the management of fracture nonunions. However, this was beyond the scope of this study, which focused on the analysis of the mussels' hard tissue after exposure to rESWs. A third limitation was that only one time point after exposure to rESWs was investigated. However, this may be of limited importance considering that, in the treatment of fracture nonunions with ESWT, treatment success is considered as evidence of radiographic union six months after the end of ESW treatment.

## Conclusions

As in the application of fESWs, induction of biomineralization in hard tissue by exposure to rESWs may not be restricted to the region of direct energy transfer into calcified tissue. Furthermore, the results of this study may contribute to better understand why the application of higher EFDs beyond a certain threshold does not necessarily lead to higher success rates when treating fracture nonunions with ESWT.

## Data Availability

The datasets used and analyzed in this study are available from the corresponding author on reasonable request.

## Abbreviations

EFD: Energy flux density
ESWs: Extracorporeal shock waves
ESWT: Extracorporeal shock wave therapy
fESWs: Focused extracorporeal shock waves
fESWT: Focused extracorporeal shock wave therapy
µs: Microsecond
rESWs: Radial extracorporeal shock waves
rESWT: Radial extracorporeal shock wave therapy
